# Hand injuries in Kenya: a chaff cutter menace

**DOI:** 10.12688/f1000research.126590.1

**Published:** 2022-11-04

**Authors:** Samuel Wanjara, Peter Oduor

**Affiliations:** 1 Department of Surgery, Nakuru Level V Hospital, Nakuru, Kenya; 2 Department of Surgery, Egerton University, Nakuru, Kenya

**Keywords:** Hand injuries, chaff-cutter, Kenya, return-to-work

## Abstract

Introduction

Hand injuries are a recognized occupational hazard from the use of chaff cutters. With increasing mechanization of farming in our region, the burden of hand injuries is poised to increase.

Methods

We conducted a descriptive study of 47 patients presenting with chaff cutter hand injuries at our center in one year.

Results

They were predominantly male (98%) and the majority (70%) were aged between 21 and 40 years. The majority of patients who had not resumed work were those with severe injuries and those who had had an amputation. There was a positive correlation between age category and severity of injury.

Discussion

Chaff cutter injuries contribute considerably to hand amputations at our center. The majority of patients with severe injuries and those undergoing amputations do not return to gainful activities one year after sustaining the injury, suggesting increased dependency. Further research is paramount to identify safety features of chaff cutters in this region.

## Introduction

Agriculture is among the most hazardous economic fields ( Mucci *et al.*, 2020) In dairy farming, the commonest site of injury are the extremities ( Pratt *et al.*, 1992). The hands are the most commonly affected, sustaining an injury in 20.5% of cases ( Naseer *et al.*, 2016; Mucci *et al.,* 2020). The use of farm machinery such as hay bailers and chaff cutters has been implicated as the commonest cause of hand injury ( Naseer *et al.*, 2016) Chaff cutters are used in chopping of fodder in dairy farming. Whereas the traditional chaff cutters were manually operated and safe, current cutters in use are power driven and more unsafe ( Naseer *et al.*, 2016; Ali *et al.,* 2021). Increasing mechanization predisposes workers to high risks for injuries, often with devastating outcomes ( Angoules *et al.*, 2007). Incidence of hand injuries sustained from chaff cutters vary with age and sex, with a preponderance for young male adults ( Naseer *et al.*, 2016; Das, 2014; Mucci *et al.,* 2020; Patel *et al.,* 2018).

The majority of the injuries from chaff cutters are non-fatal ( Das, 2014; He *et al.,* 2010; Patel *et al.,* 2018); however, they can lead to limb loss and loss of function ( Wairegi, 2019). They vary from simple lacerations, dislocations, fractures, crush injuries and amputations. Campbell and Kay proposed an objective grading system, the Hand Injury Severity Score (HISS), for the severity of the injuries to enhance assessment, communication and plan of management with consideration of the skin, motor, neural and skeletal components of the injury ( Naseer *et al.*, 2016; Campbell & Kay, 1996). The hand is an important organ, especially in low-income settings, where it is thought to create an identity for the individual in the society ( Naseer *et al.*, 2016). Moreover, hand injuries resulting from chaff cutters are more likely to involve the dominant hand thereby affecting the capabilities of the affected in participating in gainful activities and in integrating in the society ( Naseer *et al.*, 2016; Wairegi, 2019). This leads to social and economic losses until the individuals are fit to return to pre-injury activities ( Ali *et al.,* 2021). The return to gainful activities and social incorporation after injury is influenced by demographic, clinical and economic factors ( He *et al.,* 2010).

Our region, and Kenya in particular, has a robust dairy industry that is experiencing rapid commercialization, mechanization and innovation ( Bebe *et al.,* 2015; Mutavi, 2017). With the increased mechanization of farming in the region, the burden of hand injuries is poised to increase. However, literature on characteristics and management of these injuries from our region is largely limited. We therefore aimed to study the characteristics of chaff cutter injuries from our center, including their management and impact on return to gainful activities.

## Methods

We conducted a retrospective descriptive study on patients presenting with chaff cutter hand injuries. The study was done in Nakuru County, in Kenya. We collected data for all patients who presented and were managed from Nakuru Level V Hospital during the period starting July 2019 through June 2020. Patients who did not give an informed written consent for participation were excluded. The case notes for the patients were retrieved from patients’ records and from the register of operations in the operating room. The patients’ biodata, type of injury and the surgical procedure performed were obtained. Self-reported sex category was obtained retrospectively from records of routine data in our unit. We included all injuries in the volar zones of the hand(s) distal to the wrist joint and assigned a severity score for the following categories; integument, skeletal, motor and neural. The individual category scores were aggregated into a summative Hand Injury Severity Score (HISS) score for every patient.

Subsequently, an enquiry was then made through a telephone call to the patients or their caretakers, a year after the month of hospital discharge, collecting data on whether the patient had resumed gainful activity. Data was entered into Microsoft Excel ( *Microsoft Corp*) and exported for analysis.

### Data analysis

The data were analyzed using Stata Version 14, ( *StataCorp LLC Canada, US)* **.** Fischer’s exact test was used to assess the relationship of resumption to work and surgical procedure performed while Pairwise correlation was used to test for correlation between age and sex with severity of injury. A p-value of ≤0.05 was considered statistically significant where applicable.

### Ethical review

We obtained ethical review exemption from Nakuru Level V Hospital ethical committee (Ref: ERC/NLV5/2021/9) in consideration of the observational design of our study. In addition, we only intended to utilize anonymized routine clinical data.

## Results

Forty-seven patients were included. The age varied from 14 to 69 years with the mean age being years 28.34 (SD 11.91) with a median of 25 years. They were predominantly male (98%) while the majority (70%) were aged between 21 and 40 years.

**Figure 1.**
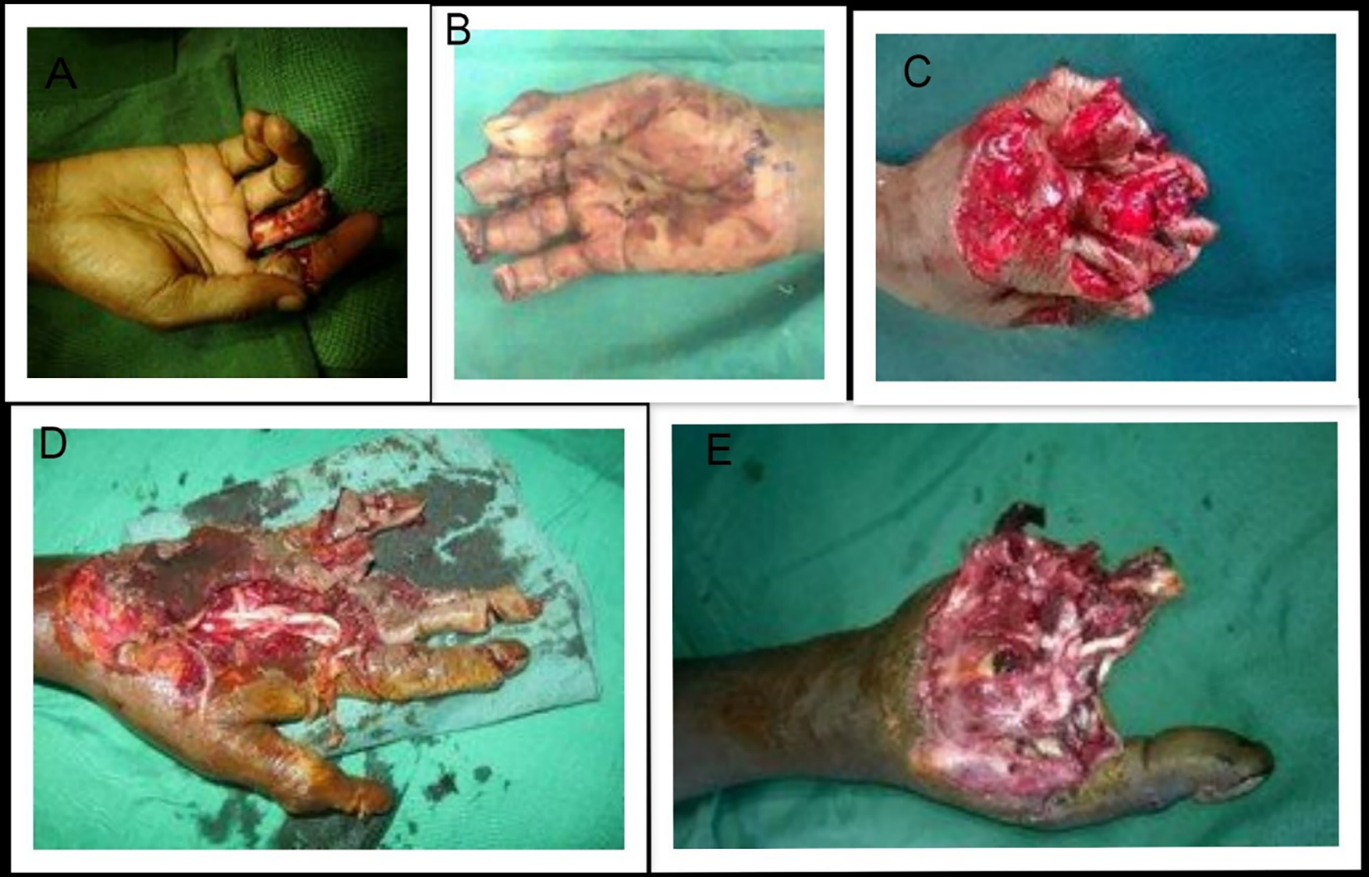
This figure demonstrates examples of various patterns of chaff cutter hand injuries (A-E); A: One finger injury; B: four finger traumatic amputation; C-E: Combined crush and tendon injuries.

Hand injuries were assessed and graded with the HISS. Injuries with a HISS score of above 51 were grouped as severe/major injuries, while those with a HISS score of 21–50 were graded as moderate and HISS score below 20 as minor. 36.2% of the patients equally had minor and severe/major injuries while 27.7% had moderate injuries ( Figure 1). The majority of patients with severe injuries were between 21 and 40 years (58.8%), and all of them underwent an amputation. Many of the patients with minor injuries underwent debridement alone (41.2%) while the rest had either debridement with repair (23.5%) or primary amputation (35.3%). Table 1 demonstrates the HISS scores.

**Table 1.** This table demonstrates the Hand Injury Severity grading.

		Injury Severity				
		Minor	Moderate	Sever/Major	Total	P Value
		n=17 (36.2%)	n=13 (27.7%)	n=17 (36.2%)	N=47	
Sex n (%)	Male	17 (37.0)	13 (28.3)	16 (34.8)	(97.9)	0.41
	Female	0 (0.0)	0 (0.0)	1 (100.0)	1 (2.1)	
Age category n (%)	Less than 20 years	7 (50.0)	4 (28.6)	3 (21.4)	14 (29.8)	0.39
	21 to 40 years	9 (33.3)	8 (29.6)	10 (37.0)	27 (57.5)	
	Above 40 years	1 (16.7)	1 (16.7)	4 (66.7)	6 (12.7)	
Surgical procedure n (%)	Debridement	7 (77.8)	2 (22.2)	0 (0.0)	9 (19.1)	0.00
	Amputation	6 (18.2)	10 (30.3)	17 (51.5)	33 (70.3)	
	Repair	4 (80.0)	1 (20.0)	0 (0.0)	(10.6)	

The majority of patients who had not resumed work were those with severe injuries (60.0%) and those who had had an amputation (84.4%). Table 2 demonstrates the resumption to work. There was no statistically significant relationship between surgical procedure and resumption to work (p=0.120). There was a positive correlation between age category and severity of injury (r=0.2781, p>0.005). A negative correlation was observed for sex and severity of injury. (r=−0.1734, p>0.005) However, there was no significant association among age, sex and severity of injury.

**Table 2.** This table shows the Resumption to work against sex, age, surgical procedure performed and severity of the injury.

Attribute		Resumption to work				
		Yes	No	Lost to follow up	Total	P Value
		n=21 (44.7%)	n=25 (53.2%)	n=1,2.1%)	(N=47)	
Sex n (%)	Male	21(45.7)	24 (52.2)	1 (2.2)	46 (97.9)	0.64
	Female	0 (0.0)	1 (100.0)	0 (0.0)	1 (2.1)	
Age category n (%)	Less than 20 years	6 (42.9)	8 (57.1)	0 (0.0)	14 (29.8)	0.47
	21 to 40 years	14 (51.9)	12 (44.4)	1 (3.7)	27 (57.5)	
	Above 40 years	1 (16.7)	5 (83.3)	0 (0.0)	6 (12.7)	
Surgical procedure n (%)	Debridement	7 (77.8)	2 (22.2)	0 (0.0)	9 (19.1)	0.18
	Amputation	11 (33.3)	21 (63.6)	1 (3.0)	33 (70.3)	
	Repair	3 (60.0)	2 (40.0)	0 (0.0)	5 (10.6)	
Severity of injury n (%)	Minor	12 (70.6)	5 (29.4)	0 (0.0)	17 (36.2)	0.00
	Moderate	8 (61.5)	5 (38.5)	0 (0.0)	13 (27.6)	
	Severe/Major	1 (5.9)	15 (88.2)	1 (5.9)	17 (36.2)	

## Discussion

The findings from our study showed that male persons aged between 21 and 40 years were the commonest group of workers likely to sustain chaff cutter injuries involving the hand. In a study from Pakistan including 30 cases of *tokka* (chaff cutter) injuries, the male-to-female ratio of the victims was 3:1 ( Ali *et al.,* 2021). In contrast, in another retrospective study from Pakistan, Naseer *et al.* (2016) found that male persons were affected in only 43.8% of the cases. The likely explanation for this observation was that fodder chopping was considered a domestic chore in the study’s cultural background and likely to be performed by women and children ( Naseer *et al.*, 2016). Similarly in industrialized nations, machine injury accounts for the majority of injuries to the hand ( Naseer *et al.*, 2016; Cooper *et al.,* 2006). A systematic review of upper limb injuries in agriculture by Mucci and colleagues found that the 20 to 45 year old age group was the most affected by chaff cutter injuries, agreeing with our findings. They observed that this could be explained by a workforce dominated by the young, in low- and high-income settings alike ( Mucci *et al.,* 2020).

In a study from Nepal on hand injuries caused by fodder cutters, Kishor and colleagues demonstrated that majority of the injuries were minor to moderate ( Shrestha *et al.,* 2018). In our study, no degree of injury predominated significantly. Wairegi further notes that in patients who underwent upper limb amputations from a tertiary hospital in Kenya, the majority of the injuries were from chaff cutters ( Wairegi, 2019). The severity of hand injuries could be dependent on age, literacy levels and time of day ( Angoules *et al.,* 2007; Wairegi, 2019). We found a positive correlation between age and severity of injury. In addition, patients aged between 21 and 40 years were more likely to sustain a severe injury. This could also be explained by the fact that majority of those injured were young and that the workforce in agriculture in low income countries is also predominantly young ( Mucci *et al.*, 2020). Work by Mucci and colleagues suggested that individuals with low literacy levels had a higher risk of injury than their counterparts with higher educational status ( Mucci *et al.,* 2020). However, our study did not investigate the impact of educational status.

The return to gainful activities after injury is hugely affected by the severity of injury ( He *et al.,* 2010). At one year after the injury, majority of patients in our study had not returned to their pre-injury occupations with the majority being those with severe/major injuries. On the contrary, Savitsky *et al.* observed that the period to return to work among individuals injured in the work setting was independent of injury characteristics ( Savitsky *et al.,* 2020). However, their findings might have been affected by the incentive of occupational injury allowances given to participants in their study. Notably, in our setting injured patients do not receive any such allowances, which potentially impoverishes them further. In general, farm injuries lead to profound physical and emotional disability and economic loss ( Angoules *et al.,* 2007). The period taken to return to work might be an indicator of temporary or permanent disablement, suggesting inability of the affected individual to earn a livelihood during this period and to lose their social standing. This consequently makes them depend on other persons for sustenance ( Naseer *et al.*, 2016; Savitsky *et al.,* 2020).

Prevention of farmhand injuries is an key priority to avoid the economic loss, reduction of quality of life and emotional and social alterations resulting from such injuries ( Angoules *et al.,* 2007). Specific interventions, legislative or otherwise, ought to be designed to achieve practical safety standards ( Voaklander *et al.,* 2006). Das states that the majority of these injuries result from human factors (73%), with machine factors contributing to only 13% ( Das, 2014). Imperatively, therefore, efforts by governments and/or employers should be concentrated on worker education especially on occupational health and safety ( Naseer *et al.*, 2016). In addition, a design change should be strongly considered to guarantee safety ( Kumar *et al.,* 2013; Paul, 2012).

Our study had several limitations. First, we did not assess the handedness of the subjects. This would have enabled us to determine if there was any association between handedness with severity of injury or with the period taken to resume work. Secondly, our study had a relatively small sample size which weakens the strength of our findings and potentially affects the generalizability of our conclusions. Lastly, the design of our study limited the assessment of the possible effect of gender differences on the patterns of injury, treatment offered or on the period of return to gainful activities. The proportion of female sex was also low in our study - this might adversely affect the generalizability of our findings. Nevertheless, we are confident that the insights from our work will raise awareness on this problem, avail the much-needed literature from our region and hopefully inform interventions to curb the chaff cutter menace.

In conclusion, chaff cutter injuries contribute considerably to hand amputations at our center. The majority of patients with severe injuries and those undergoing amputations do not return to gainful activities one year after sustaining the injury. Further research is needed into the safety features of chaff cutters in use in Nakuru county to help reduce such injuries. The prolonged interval to return to work might also suggest increased dependency at a young age.

## Data Availability

Figshare: Chaff Cutter Hand Injuries from Nakuru Kenya. https://doi.org/10.6084/m9.figshare.21213293.v1 ( Wanjara and Oduor, 2022). This project contains the following underlying data:
•
Chaff Cutter Hand Injuries from Nakuru Kenya.xlsx. (Data on baseline characteristics, injury pattern and severity, and period to return to work) Data are available under the terms of the Creative Commons Zero “No rights reserved” data waiver (CC0 4.0 Public domain dedication).
