# Supplementary material for: Hand injuries in Kenya: a chaff cutter menace
Source: F1000Res. 2022 Nov 4;11:1255. [Version 1] doi: 10.12688/f1000research.126590.1 (PMC10620478; doi:10.12688/f1000research.126590.1)
Supplement: Peer review history

# Reviewer report: Reviewer response for version 1

**DOI:** 10.5256/f1000research.139019.r216316

**Published:** 2023-11-01

**Authors:** Mahmoud A Hifny

**Affiliations:** 1 Plastic Surgery Department, Faculty of Medicine, Qena University Hospital, South Valley University, Qena, Qena Governorate, Egypt

Thank you so much for your kind invitation to review this article entitled “Hand Injuries in Kenya: a chaff cutter menace“

 In this research, the authors conducted a descriptive study of 47 patients presenting with chaff cutter hand injuries at their center over a one year period. They found that the majority of patients with severe injuries according to the HISS score were between 21 and 40 years (58.8%), and all of them underwent an amputation and many of them had not resumed their work again. The authors claimed that chaff cutter injuries contribute considerably to hand amputations and it is paramount to identify the safety features of using chaff cutters in their region in order to reduce such injuries.

 This article is well-written with a nice flow and relevant information. The topic is of great significance given the increased frequency of hand injuries following increasing mechanization of the chaff cutter which predisposes workers to high risks for injuries with devastating outcomes.

 Although the authors represent a good descriptive retrospective study with an excellent review of the previous literature in their discussion, I have a few minor issues with regard to this study.

 The authors stated that “We found a positive correlation between age and severity of injury”. Could you describe an explanation of this correlation in your opinion?

 Also the authors reported that “At one year after the injury, majority of patients in our study had not returned to their pre-injury occupations with the majority being those with severe/major injuries”. It would be better to add a percentage instead of the word ‘majority’

 I feel that it is appropriate to discuss the author’s recommendations, from their point of view, the safety measures that should be applied when using the chaff cutter in order to reduce the incidence of such injuries. Or it would be better that the authors cite other researchers who described the safety measures to minimize the hazards of using this machine but the researchers.

Is the work clearly and accurately presented and does it cite the current literature?

Yes

If applicable, is the statistical analysis and its interpretation appropriate?

I cannot comment. A qualified statistician is required.

Are all the source data underlying the results available to ensure full reproducibility?

Yes

Is the study design appropriate and is the work technically sound?

Yes

Are the conclusions drawn adequately supported by the results?

Partly

Are sufficient details of methods and analysis provided to allow replication by others?

Yes

Reviewer Expertise:

Plastic Surgery, Hand Surgery, Microsurgery, Burn, Reconstructive Surgery

I confirm that I have read this submission and believe that I have an appropriate level of expertise to confirm that it is of an acceptable scientific standard, however I have significant reservations, as outlined above.

# Reviewer report: Reviewer response for version 1

**DOI:** 10.5256/f1000research.139019.r216314

**Published:** 2023-10-31

**Authors:** Salah Fuad Issa

**Affiliations:** 1 University of Illinois at Urbana-Champaign, Urbana, USA

This paper evaluated chaff or fodder cutter injuries in a hospital in Kenya. It followed up with 47 individuals who received injuries due to fodder cutters and found that the majority where male between ages 20-40 years old and those who received severe injuries (amputations) were more likely not to return to work a year after the injury. This study builds on existing reports in Pakistan and other countries on the dangers of fodder cutters.

 Describe the reasons you used different statistical methods to calculate correlation.

 In figure 1, I would add what each injury represents in terms of HISS score. For example, figure A is graded with a HISS score below 20 and so on.

 Table 1 needs rework, you are using brackets as % for rows, but when you reach the total it becomes across category, it is a bit hard to follow. Also missing some values in the table (example total male incidents).

 For surgical procedure and injury level in table 1, what method did you use to calculate p-value, not clear from methods.

Is the work clearly and accurately presented and does it cite the current literature?

Yes

If applicable, is the statistical analysis and its interpretation appropriate?

I cannot comment. A qualified statistician is required.

Are all the source data underlying the results available to ensure full reproducibility?

Yes

Is the study design appropriate and is the work technically sound?

Yes

Are the conclusions drawn adequately supported by the results?

Yes

Are sufficient details of methods and analysis provided to allow replication by others?

Partly

Reviewer Expertise:

Agricultural safety, machine safety, grain safety

I confirm that I have read this submission and believe that I have an appropriate level of expertise to confirm that it is of an acceptable scientific standard, however I have significant reservations, as outlined above.

# Reviewer report: Reviewer response for version 1

**DOI:** 10.5256/f1000research.139019.r157449

**Published:** 2022-12-20

**Authors:** Joseph Wanjeri

**Affiliations:** 1 University of Nairobi, Nairobi, Kenya

This is a good article based on the results of a descriptive retrospective study. The title is however misleading because the study was conducted at only one hospital/institution in Kenya and the results therefore may not apply to the whole country. In other words, the sample is not representative of the whole population of Kenya.

 There are a few grammatical errors including two instances of incorrect word use: Instead of ‘poised’ the authors could have used ‘are likely’ and in place of ‘paramount’ they should have simply used ‘needed’. There are also instances where sentences needed to be merged to improve the grammar. I appreciate however that English is not the authors’ first language.

 This was a descriptive retrospective study but the researchers took liberty to apply analytic statistics which I feel was inappropriate and as a result of it some of the inferences and conclusions can be challenged. However, in their discussion the researchers do acknowledge the study limitations which is a good thing.

 Concerning the recommendation, the authors cite other researchers who reported that human/user/operator factors were more important in chaff cutter injuries than machine/equipment factors. It would have been better to recommend that further research is needed to determine the risk factors for chaff cutter injuries seen at Nakuru Level 5 Hospital.

 All in all this was a good seminal research for Nakuru county in Kenya and the report of which will facilitate future research.

Is the work clearly and accurately presented and does it cite the current literature?

Yes

If applicable, is the statistical analysis and its interpretation appropriate?

Partly

Are all the source data underlying the results available to ensure full reproducibility?

Yes

Is the study design appropriate and is the work technically sound?

Yes

Are the conclusions drawn adequately supported by the results?

No

Are sufficient details of methods and analysis provided to allow replication by others?

Yes

Reviewer Expertise:

Plastic and Reconstructive Surgery, Research Methodology. Research interest: Burn injuries

I confirm that I have read this submission and believe that I have an appropriate level of expertise to confirm that it is of an acceptable scientific standard.
